# Calorie-induced ER stress suppresses uroguanylin satiety signaling in diet-induced obesity

**DOI:** 10.1038/nutd.2016.18

**Published:** 2016-05-23

**Authors:** G W Kim, J E Lin, A E Snook, A S Aing, D J Merlino, P Li, S A Waldman

**Affiliations:** 1 Department of Pharmacology and Experimental Therapeutics, Thomas Jefferson University, Philadelphia, PA, USA; 2 Department of Pathology, Stanford University, Stanford, CA, USA

## Abstract

**Background/Objectives:**

The uroguanylin-GUCY2C gut–brain axis has emerged as one component regulating feeding, energy homeostasis, body mass and metabolism. Here, we explore a role for this axis in mechanisms underlying diet-induced obesity (DIO).

**Subjects/Methods:**

Intestinal uroguanylin expression and secretion, and hypothalamic GUCY2C expression and anorexigenic signaling, were quantified in mice on high-calorie diets for 14 weeks. The role of endoplasmic reticulum (ER) stress in suppressing uroguanylin in DIO was explored using tunicamycin, an inducer of ER stress, and tauroursodeoxycholic acid (TUDCA), a chemical chaperone that inhibits ER stress. The impact of consumed calories on uroguanylin expression was explored by dietary manipulation. The role of uroguanylin in mechanisms underlying obesity was examined using *Camk2a-Cre-ER*^*T2*^-*Rosa-STOP*^*loxP/loxP*^*-Guca2b* mice in which tamoxifen induces transgenic hormone expression in brain.

**Results:**

DIO suppressed intestinal uroguanylin expression and eliminated its postprandial secretion into the circulation. DIO suppressed uroguanylin through ER stress, an effect mimicked by tunicamycin and blocked by TUDCA. Hormone suppression by DIO reflected consumed calories, rather than the pathophysiological milieu of obesity, as a diet high in calories from carbohydrates suppressed uroguanylin in lean mice, whereas calorie restriction restored uroguanylin in obese mice. However, hypothalamic GUCY2C, enriched in the arcuate nucleus, produced anorexigenic signals mediating satiety upon exogenous agonist administration, and DIO did not impair these responses. Uroguanylin replacement by transgenic expression in brain repaired the hormone insufficiency and reconstituted satiety responses opposing DIO and its associated comorbidities, including visceral adiposity, glucose intolerance and hepatic steatosis.

**Conclusions:**

These studies reveal a novel pathophysiological mechanism contributing to obesity in which calorie-induced suppression of intestinal uroguanylin impairs hypothalamic mechanisms regulating food consumption through loss of anorexigenic endocrine signaling. The correlative therapeutic paradigm suggests that, in the context of hormone insufficiency with preservation of receptor sensitivity, obesity may be prevented or treated by GUCY2C hormone replacement.

## Introduction

Obesity is a global pandemic and >1.5 billion adults are overweight (body mass index >25 kg m^−2^), 500 million of whom are obese (body mass index >30 kg m^−2^).^1, 2, 3^ Within the United States, 69% of adults are overweight and 35% are obese, a figure that has doubled over 20 years.^4^ The US health-care costs of obesity are $147 billion, and within 15 years 20% of health-care costs will reflect chronic diseases of obesity.^2, 3, 4, 5^ These observations underscore the unmet need for new management paradigms for obesity and its comorbidities.

The hormone uroguanylin, whose expression is limited to small intestine, has emerged as one component of a gut–brain axis regulating feeding, energy homeostasis, body mass and metabolism.^6, 7, 8^ Food consumption induces secretion of prouroguanylin from the small intestine into the circulation in mice and humans, with delivery to the hypothalamus.^8^ There, the propeptide is proteolyzed to mature uroguanylin, where it interacts with its only known receptor, GUCY2C, one of the cyclic guanosine monophosphate (cGMP)-producing receptors.^8^ Indeed, intravenous (i.v.) or intracerebroventricular (i.c.v.) injection of GUCY2C ligands induce satiety in wild-type (*Gucy2c*^*+/+*^) mice^7, 8^ but not in GUCY2C-deficient (*Gucy2c*^*-/-*^) mice.^8^ Beyond these anorexigenic responses, an effect of i.c.v. uroguanylin on fecal output and the generation of brown adipose tissue and energy expenditure has been suggested.^7^ Together, these uroguanylin responses oppose the development of diet-induced obesity (DIO) and the metabolic syndrome.^6, 7, 8^

Although a contribution of the uroguanylin gut–brain axis to the complex endocrine regulation of energy homeostasis and body mass has emerged, a role for this axis in the pathophysiology of obesity has not yet been explored. The present studies examine a novel pathophysiological mechanism contributing to obesity in which calorie-induced suppression of intestinal uroguanylin silences hypothalamic GUCY2C regulating satiety.

## Materials and methods

### Animals

All studies were conducted under protocols approved by the institutional animal care and use committee. C57BL/6J (000664), *ob/ob* (000632) and *Camk2a-Cre-ER*^*T2*^ (012362) mice were obtained from The Jackson Laboratory (Bar Harbor, ME, USA). Animals were housed in the Laboratory Animal Facility at Thomas Jefferson University under conditions approved by the institutional animal care and use committee, including a 12 h diurnal light/dark cycle, constant temperature (68–74 °F) and continuous access to food and water, unless otherwise noted. Generally, mice were housed 3–5 in a cage, except for studies of daily food intake, where they were housed in individual wire-mesh cages.^8^

*Gucy2c*^*–/–*^ mice^9^ were backcrossed with C57BL/6J mice for more than 10 generations to produce *Gucy2c*^*–/–*^ congenic C57BL/6J mice and wild-type (*Gucy2c*^*+/+*^) littermates. Only colony-bred *Gucy2c*^*+/+*^ mice were compared with *Gucy2c*^*–/–*^ mice. These mice were used for studies of the effects of GUCY2C ligand on *c-Fos* and *Pomc* expression.

For *Rosa-STOP*^*loxP/loxP*^*-Guca2b* mice, the *Guca2b* construct consisted of the *Rosa26* promoter sequence, a STOP cassette flanked by two *loxP* sites and full-length *Guca2b*. Conditional expression in brain was selected to examine the effects of uroguanylin on local hypothalamic, rather than distal enterocyte, GUCY2C without the necessity of exogenous peptide delivery. Thus, *Camk2a-Cre-ER*^*T2*^ mice were bred with *Rosa-STOP*^*loxP/loxP*^*-Guca2b* mice to produce hemizygous *Camk2a-Cre-ER*^*T2*^-*Rosa-STOP*^*loxP/loxP*^*-Guca2b* mice. *Camk2a-Cre-ER*^*T2*^ mice were bred with hemizygous *Camk2a-Cre-ER*^*T2*^-*Rosa-STOP*^*loxP/loxP*^*-Guca2b* mice to produce *Camk2a-Cre-ER*^*T2*^-*Rosa-STOP*^*loxP/loxP*^*-Guca2b* mice and littermate controls lacking the *Rosa-STOP*^*loxP/loxP*^*-Guca2b* transgene. Tamoxifen (Sigma-Aldrich, St Louis, MO, USA) was dissolved in sunflower seed oil (Sigma-Aldrich) and administered by intraperitoneal (i.p.) injection (100 mg kg^−1^ per day) for 5 days^10^ every 6 weeks to delete the floxed STOP cassette and express Guca2b. These mice were used for studies of the effects of transgenic uroguanylin replacement on appetite regulation.

### Adiposity

Fat was dissected from subcutaneous (hind, interscapular) and visceral (epididymal, mesenteric, perineal, retroperitoneal) fat pads and weighed.^8^

### Chicken anti-uroguanylin antibody

A polyclonal chicken antibody against prouroguanylin (PU0247) was produced by Thermo Scientific (Waltham, MA, USA). The antigenic peptide, QLESVKKLNELEEKEMSNPQ, was conjugated to bovine serum albumin, injected with Freund's complete adjuvant for primary inoculation or with Freund's incomplete adjuvant for two subsequent boosts. The peptide was conjugated to Blue Carrier (Thermo), and injected with Freund's complete or incomplete adjuvant in two final boosts. IgY antibodies were purified from egg yolks and validated by enzyme-linked immunosorbent assay.

### Daily food intake

Mice were acclimated to individual cages with wire-mesh floors for 1 week.^8^ Food consumption was measured daily for 10 days and averaged for each animal.

### Diets

Rodent Diet 5010 (LabDiet, St Louis, MO, USA) is a low-calorie, standard-chow (lean) diet (3.1 kcal g^−1^, 12.7% calories from fat, 58.5% calories from carbohydrates and 28.8% calories from protein); Diet 58Y1 is a high-calorie, high-fat diet (5.1 kcal g^−1^, 61.6% calories from fat, 20.3% calories from carbohydrates and 18.1% calories from protein); and Diet 58Y2 is a moderate-calorie, high-carbohydrate diet (3.8 kcal g^−1^, 10.2% calories from fat, 71.8% calories from carbohydrate and 18.0% calories from protein; Supplementary Table S1). Mice were maintained on diets *ad libitum* from 6 to 20 weeks of age. In studies of reversible uroguanylin loss, mice were either maintained on Diet 5010 or Diet 58Y1 for 18 weeks, or placed on Diet 58Y1 for 14 weeks and then switched back to Diet 5010 for 4 weeks. In studies with the *ob/ob* strain, mice were either allowed *ad libitum* feeding or restricted to 3 g day of Diet 5010 for 6 weeks.

### Enzyme-linked immunosorbent assay

Mice were fasted for 16 h overnight, blood was collected before and after 1 h of refeeding and serum was isolated. A rabbit anti-prouroguanylin antibody, 6912 (M Goy, University of North Carolina, Chapel Hill, NC, USA^11^), was used (1:1000) to coat Nunc-Immuno PolySorp plates (Thermo) for 16 h at 4 °C. The prouroguanylin peptide (CQQKSGLLPDVSYNP) was serially diluted (1:5) in Superblock T20 (phosphate-buffered saline) Blocking Buffer (Thermo) and plated to generate a standard curve (1 μg ml^−1^ to 2.56 pg ml^−1^). Serum samples were diluted (1:10) in Superblock and plated. The peptide was biotinylated using EZ-Link Sulfo-NHS-Biotin (Thermo), diluted in Superblock and added to each standard and sample. Plates were sequentially incubated for 30 min at 37 °C, with streptavidin-horseradish peroxidase (Thermo) for 30 min at 37 °C, with 1-Step Turbo TMB-ELISA Substrate Solution (Thermo) for 30 min at room temperature, stopped with 1 M H_3_PO_4_ and absorbance quantified at 460 nm.

### Endoplasmic reticulum (ER) stress

In some studies, mice were i.p. injected for 5 days with vehicle (1% dimethyl sulfoxide in phosphate-buffered saline) or 1 mg kg^−1^ per day of tunicamycin (Sigma-Aldrich) to induce ER stress. In other studies, mice were orally gavaged for 12 days with vehicle (1% dimethyl sulfoxide in phosphate-buffered saline) or 150 mg kg^−1^ per day of tauroursodeoxycholic acid (TUDCA; Sigma-Aldrich) to relieve ER stress.

### Hepatic steatosis

Livers were fixed with formalin for 24 h, embedded in paraffin, sectioned to 5 μm and stained with hematoxylin and eosin. Hepatic steatosis was quantified by a blinded veterinary pathologist.^12^

### Hypothalamic microdissection

Hypothalamic nuclei were microdissected using a stereomicroscope according to stereotaxic coordinates.^13^

### Immunoblot analyses

Antibodies for immunoblot analyses included the following: BiP, NPY, phospho-VASP^Ser239^, phospho-VASP^Ser157^, β-actin, β-tubulin, glyceraldehyde-3-phosphate dehydrogenase (GAPDH) and villin-1 (Cell Signaling Technology, Danvers, MA, USA); Gucy2c (MS20)^8^ and prouroguanylin (6910, M Goy, University of North Carolina).^11^ Horseradish peroxidase-conjugated secondary antibodies were from Santa Cruz Biotechnology (Dallas, TX, USA) or Jackson ImmunoResearch Laboratories (West Grove, PA, USA), and SuperSignal West Dura Chemiluminescent Substrate and Femto Chemiluminescent Substrate were from Thermo. Band intensities were quantified by densitometry and normalized to β-actin, β-tubulin, GAPDH or villin-1.

### Immunostaining

Jejunum was fixed with formalin for 24 h, embedded in paraffin, sectioned to 5 μm, mounted on glass slides, deparaffinized, rehydrated, subjected to heat-induced antigen retrieval in 10 mM sodium citrate, pH 8.5, blocked with 5% milk and 1% normal donkey serum (Jackson ImmunoResearch Laboratories) in phosphate-buffered saline with Tween-20 for 1 h, incubated with a chicken anti-uroguanylin antibody (PU0247) and a rabbit anti-β-catenin antibody (Cell Signaling Technology) for 16 h overnight at 4 °C, incubated with fluorescently labeled anti-chicken and anti-rabbit secondary antibodies (Thermo) for 90 min, coverslipped with VECTASHIELD HardSet Mounting Medium with 4',6-diamidino-2-phenylindole (Vector Laboratories, Burlingame, CA, USA) and visualized using an EVOS FL Auto Cell Imaging System (Thermo). Adjacent sections were stained with a procedure that omitted anti-uroguanylin antibody as a negative control (Supplementary Figure S1).

### Intraperitoneal glucose tolerance test

Mice were fasted for 16 h overnight, and i.p. injected with glucose (2 g kg^−1^). Blood from the distal tail was placed on test strips 0, 15, 30, 60 and 120 min thereafter and glucose concentration quantified.

### Intravenous peptide induction in hypothalamus

Employing conditions optimized previously,^8^ mice were fasted for 16 h overnight and i.v. injected via the tail vein with 100 μl (10 μg) of the GUCY2C peptide agonist, ST (NTFYCCELCCNPACAGCY; Bachem, Torrance, CA, USA), or the inactive analog, TJU (NTFYAAELAANPAAAGAY; Bachem). For gene expression studies, hypothalamus was harvested 45 min following peptide injection,^14^ and *Fos* and *Pomc* mRNA quantified by quantitative real-time PCR.^8^ For satiety, consumption of diet 58Y1 was measured 1, 2 and 4 h following peptide injection and refeeding. For vasodilator-stimulated phosphoprotein (VASP) phosphorylation quantified by immunoblot, hypothalamus was harvested 0.5, 5, 10 or 20 min following injection.

### Reverse transcriptase-PCR

RNA was purified using the RNeasy Mini Kit (Qiagen, Valencia, CA, USA) and converted to complementary DNA using TaqMan Reverse Transcription Reagents (Applied Biosystems, Foster City, CA, USA, Thermo Scientific). The complementary DNA was subjected to quantitative real-time PCR, performed on an ABI Prism 7000 Sequence Detection System (Applied Biosystems), using TaqMan Universal PCR Master Mix (Applied Biosystems) and primer/probe sets from TaqMan Gene Expression Assays (Applied Biosystems) for: *Fos* (Mm00487425_m1), *Guca2b* (Mm01192051_m1), *Gucy2c* (Mm01267705_m1), *Npy* (Mm03048253_m1), *Pomc* (Mm00435874_m1), *Actb* (Mm01205647_g1), *Gapdh* (Mm99999915_g1) and *Vil1* (Mm00494146_m1). Relative expression was calculated with the 2^–ΔΔCT^ method, using *Actb*, *Gapdh* or *Vil1* as a reference.

### Statistical analyses

All analyses were conducted in a blinded manner. Two-tailed Student's *t*-tests were used for single comparisons, and one or two-way analysis of variance for multiple comparisons, as appropriate. Correlation of Gucy2c expression and VASP phosphorylation was analyzed by Pearson's correlation. Animal cohort sizes were calculated to be sufficient to detect two-tailed statistically significant differences with 95% confidence and 90% power, assuming unequal variances and allowing for unequal sample sizes between groups. For these studies, animals were not randomized to groups. Statistical analyses were performed with GraphPad Prism 6 software (La Jolla, CA, USA), *P*<0.05 was considered significant and data represent mean±s.e.m.

## Results

### DIO suppresses intestinal uroguanylin expression

**Figure 1:**
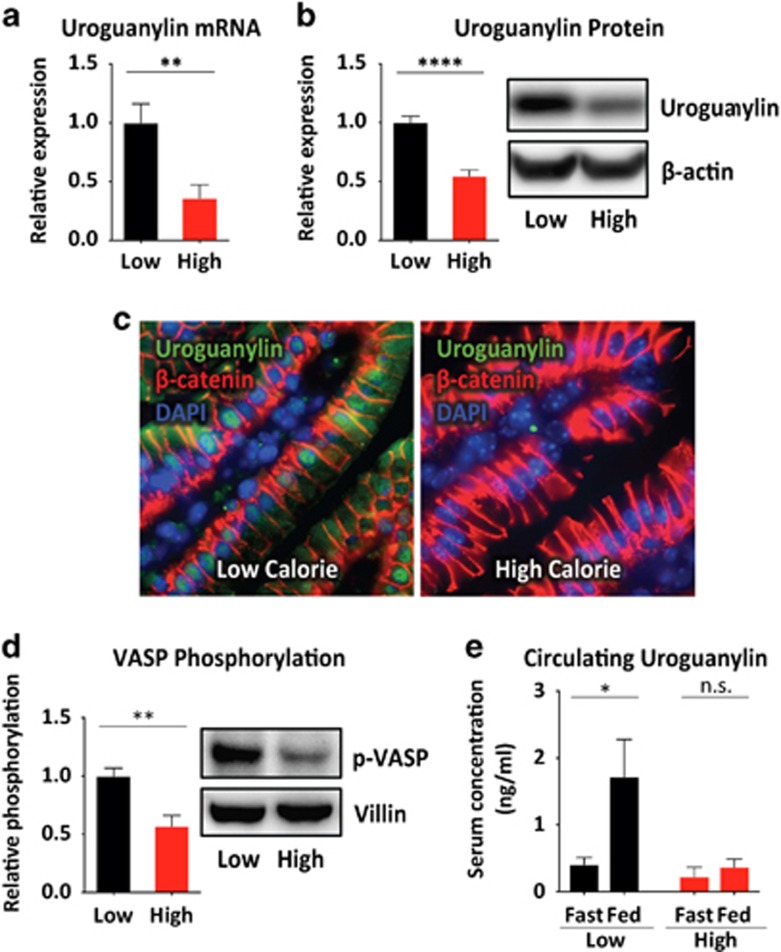
DIO induces uroguanylin loss. (**a**, **b**) Uroguanylin mRNA and protein levels in jejunal epithelium from mice maintained for 14 weeks on a low-fat (Low; *n*=8) or high-fat (High; *n*=9) diet, quantified by quantitative real-time PCR (qRT-PCR) and immunoblot and normalized to *Vil1* or β-actin expression. (**c**) Uroguanylin immunostaining (green) in jejunum from mice maintained on low- or high-fat diets. Sections were counterstained for β-catenin (red) and nuclei (blue). (**d**) VASP serine-239 phosphorylation in jejunal epithelium from mice maintained on low- or high-fat diets, quantified by immunoblot and normalized to villin-1 (*n*=4 per group). (**e**) Serum concentrations of uroguanylin in fasted and fed conditions, in mice maintained on a low- or high-fat diet, quantified by enzyme-linked immunosorbent assay (ELISA; *n*=5 per time point per group). *P<0.05, **P<0.01, ****P<0.0001; NS, not significant.

To assess the integrity of the uroguanylin-GUCY2C circuit in obesity, wild-type mice were placed on a high-fat diet to produce DIO. Uroguanylin mRNA (*P<*0.01) and protein (*P<*0.0001) in jejunum were reduced (45.4±7.5% protein reduction) after 14 weeks on the high-calorie diet, compared with lean controls on a low-calorie diet (Figures 1a–c and Supplementary Figure S1). Furthermore, DIO was associated with a commensurate reduction (43.1±11.4% *P<*0.001) in phosphorylation of VASP, a reporter of GUCY2C-cGMP signaling,^15^ in jejunal epithelium (Figure 1d). Moreover, DIO eliminated the postprandial increase in serum uroguanylin (*P<*0.05; Figure 1e).

### DIO suppresses uroguanylin by inducing intestinal ER stress

**Figure 2:**
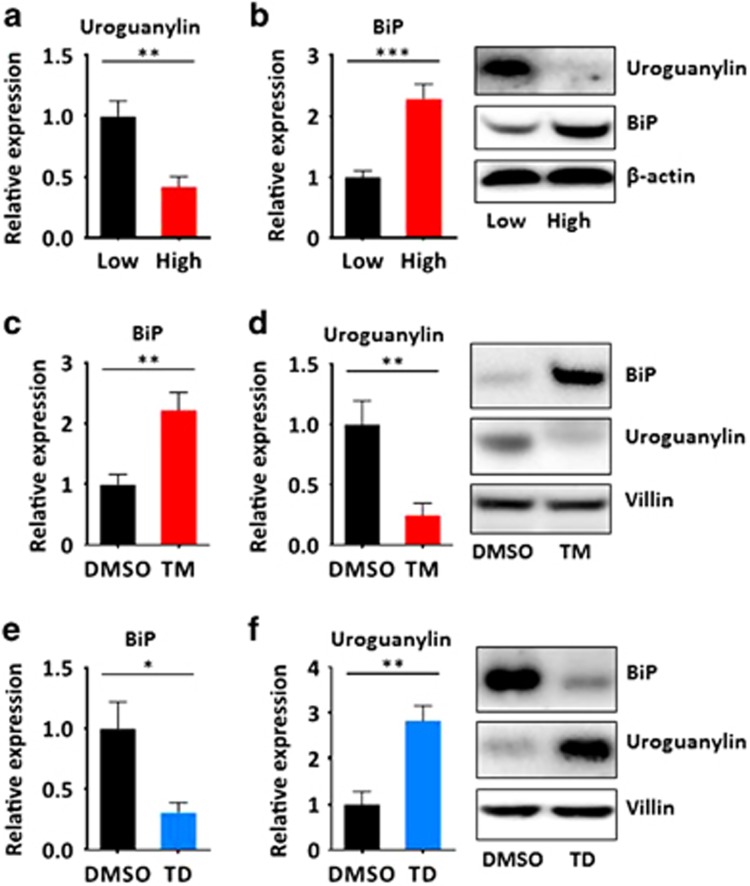
DIO induces uroguanylin loss through ER stress. Uroguanylin and BiP levels in jejunal epithelium, quantified by immunoblot and normalized to β-actin or villin-1, from mice on: (**a**, **b**) low-fat (*n*=12) or high-fat (*n*=11) diets; (**c**, **d**) the low-calorie diet, and i.p. injected for 5 days with vehicle (dimethyl sulfoxide (DMSO)) or tunicamycin (TM), 1 mg kg^−1^ per day (*n*=5 per group); (**e**, **f**) the high-calorie diet, and orally gavaged for 12 days with vehicle (DMSO) or TUDCA (TD), 150 mg kg^−1^/d (*n*=5 per group). *P<0.05, **P<0.01, ***P<0.001.

Chronic overnutrition is linked to ER stress in a variety of tissues,^16, 17, 18^ and ER stress is implicated in pathophysiological mechanisms involving metabolic hormones.^19, 20^ Here, uroguanylin loss by DIO (*P<*0.01; Figure 2a) was associated with increased expression of the canonical ER stress marker, BiP (GRP78, HSPA5),^21^ in jejunal epithelia (*P<*0.001; Figure 2b). Furthermore, induction of ER stress with i.p. tunicamycin recapitulated the effects of DIO in lean mice.^22^ Indeed, tunicamycin increased BiP (*P<*0.01; Figure 2c), confirming the induction of ER stress, associated with uroguanylin loss (*P<*0.01; Figure 2d). Conversely, relief of ER stress by oral TUDCA^22^ in obese mice on a high-fat diet reduced BiP (*P<*0.05; Figure 2e) and restored uroguanylin (*P<*0.01; Figure 2f).

### Calories mediate uroguanylin loss in DIO

**Figure 3:**
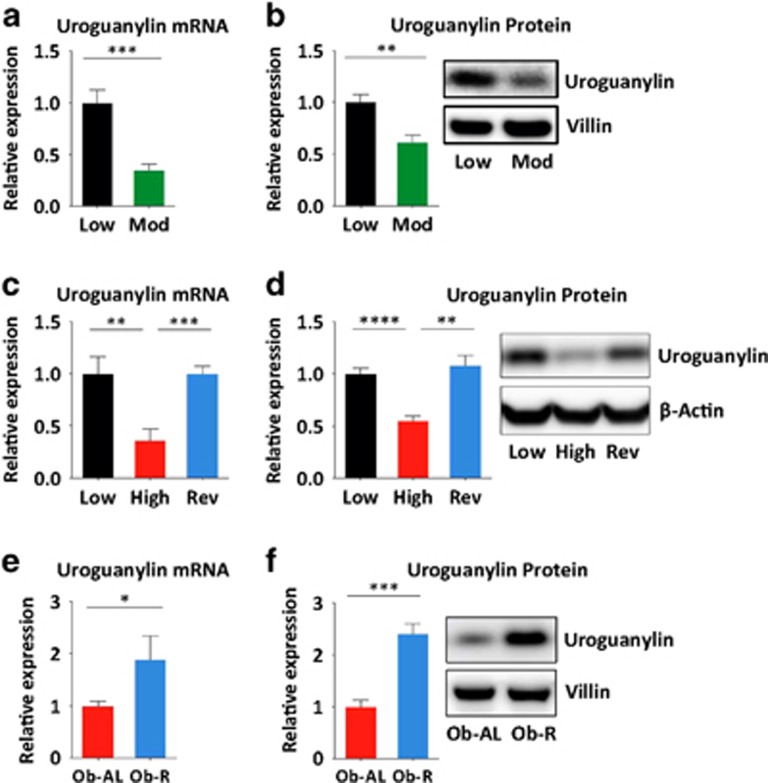
DIO-induced uroguanylin loss reflects consumed calories. (**a**, **b**) Uroguanylin mRNA and protein levels in jejunal epithelium from mice maintained on the low-calorie diet (Low; *n*=5) or a moderate-calorie, high-carbohydrate diet (Mod; *n*=9), quantified by quantitative real-time PCR (qRT-PCR) and immunoblot and normalized to *Vil1* or villin-1. (**c**, **d**) Uroguanylin mRNA and protein levels in jejunal epithelium, quantified by qRT-PCR and immunoblot and normalized to *Vil1* or β-actin from mice on: low- or high-fat diets for 18 weeks (Low, High; *n*=4 per group) or the high-fat diet for 14 weeks and then switched back to the low-fat diet for 4 weeks (Rev; *n*=8). (**e**, **f**) Uroguanylin mRNA and protein levels in jejunal epithelium from *ob/ob* mice allowed *ad libitum* feeding (Ob-AL; *n*=5) or restricted to 3 g per day for 6 weeks (Ob-R; *n*=6), quantified by qRT-PCR and immunoblot and normalized to *Vil1* or villin-1. *P<0.05, **P<0.01, ***P<0.001, ****P<0.0001.

A diet high in carbohydrates and moderately high in caloric density reduced uroguanylin mRNA (*P<*0.001; Figure 3a) and protein (*P<*0.01; Figure 3b) expression, mirroring hormone loss caused by the high-fat diet, without causing weight gain in lean mice (Supplementary Table S2). In addition, switching obese mice from a high- to a low-calorie diet restored uroguanylin mRNA (*P<*0.001; Figure 3c) and protein (*P<*0.01; Figure 3d) to normal levels even in the context of persistent DIO (Supplementary Table S2). Furthermore, restricting daily calories consumed by obese hyperphagic *ob/ob* mice to levels typical of lean mice^23^ increased uroguanylin mRNA (*P<*0.05; Figure 3e) and protein (*P<*0.001; Figure 3f) even in the context of persistent obesity (Supplementary Table S2).

### Hypothalamic GUCY2C is preserved in DIO

**Figure 4:**
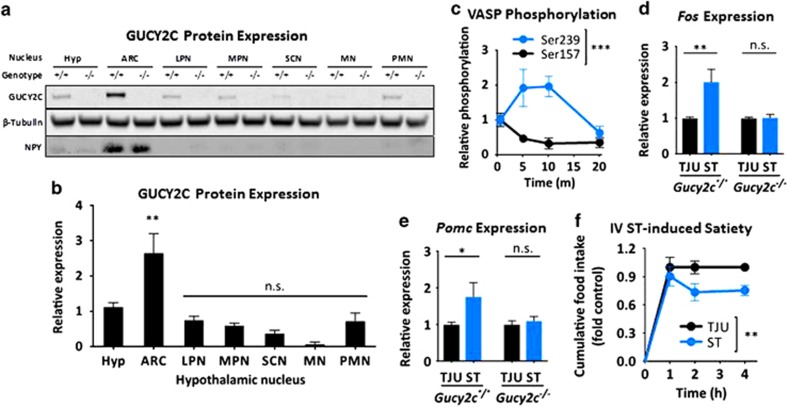
GUCY2C in the arcuate nucleus mediates anorexigenic responses to i.v. ST. (**a**, **b**) GUCY2C protein in whole hypothalamus (Hyp) and microdissected hypothalamic nuclei, quantified by immunoblot analysis and normalized to β-tubulin. Hypothalamic nuclei: ARC, arcuate nucleus; LPN, lateral preoptic nucleus; MN, mammillary nucleus; MPN, medial preoptic nucleus; PMN, premammillary nucleus; SCN, suprachiasmatic nucleus. (**c**) Hypothalamic VASP phosphorylation at serines 239 and 157 following 10 μg of i.v. ST, quantified by immunoblot and normalized to GAPDH (*n*=3 per time point per group). (**d**, **e**) Hypothalamic *Fos* and *Pomc* mRNA levels following i.v. injection with 10 μg of the negative control peptide, TJU, or ST in wild-type (*Gucy2c*^*+/+*^; *n*=18 per group) and GUCY2C-deficient (*Gucy2c*^*-/-*^; *n*=9 per group) mice, determined by quantitative real-time PCR (qRT-PCR) and normalized to *Gapdh* or *Actb*. (**f**) Food intake following 10 μg of i.v. TJU or ST (*n*=10 per group). *P<0.05, **P<0.01, ***P<0.001, NS, not significant.

**Figure 5:**
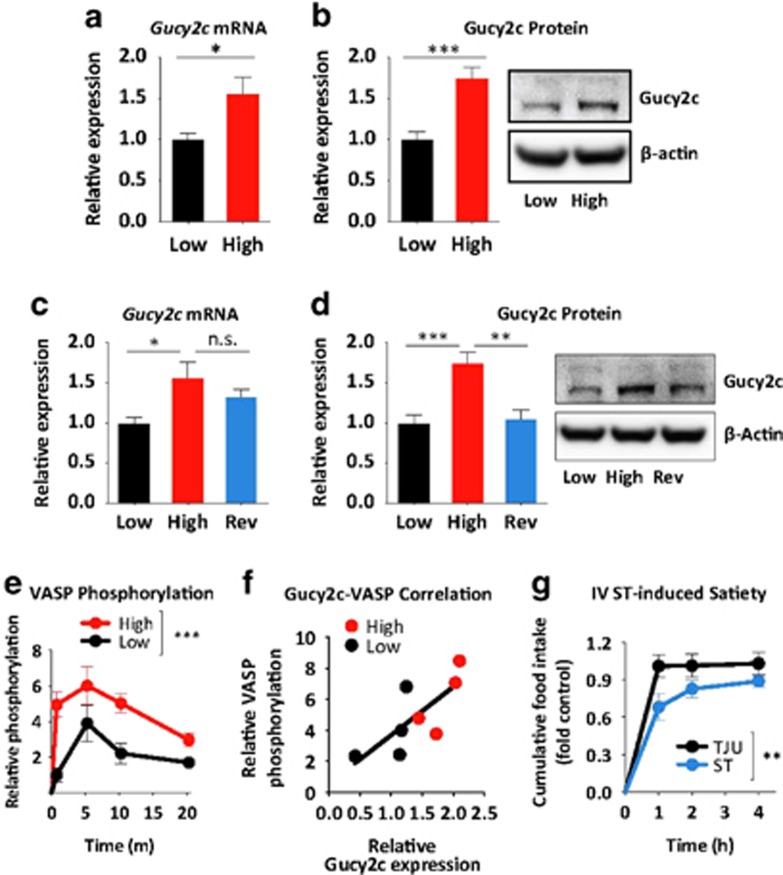
Hypothalamic GUCY2C expression and function are preserved in DIO. (**a**, **b**) Hypothalamic GUCY2C mRNA (*Gucy2c*) and protein (GUCY2C) levels in mice on low-fat (*n*=9) or high-fat (*n*=14) diets, quantified by quantitative real-time PCR (qRT-PCR) and immunoblot and normalized to *Gapdh* or β-actin. (**c**, **d**) Hypothalamic GUCY2C mRNA and protein levels in mice on Low (*n*=5), High (*n*=5) or Rev (*n*=14) diets, quantified by qRT-PCR and immunoblot and normalized to *Actb* or β-actin. (**e**) Hypothalamic VASP phosphorylation at serine 239 following 10 μg of i.v. ST in mice on low- or high-fat diets, quantified by immunoblot and normalized to β-actin (*n*=4 per time point per group). (**f**) Correlation of hypothalamic GUCY2C levels and i.v. ST-induced hypothalamic VASP phosphorylation in mice on low- or high-fat diets (*r*^2^=0.5713, *P*<0.05). VASP phosphorylation values are the levels achieved 5 min after ST injection, the peak response for both groups. The black line represents a linear regression model. (**g**) Food intake following 10 μg of i.v. TJU or ST in mice on the high-calorie diet (*n*=10 per group). *P<0.05, **P<0.01, ***P<0.001, NS, not significant.

GUCY2C expression was enriched in the arcuate nucleus (ARC; Figures 4a and b), identified by expression of NPY and POMC (Supplementary Figure S2),^24, 25, 26, 27, 28^ a key hypothalamic nucleus regulating metabolic homeostasis^29, 30^ and a central target for peripheral energy signals.^31^ The i.v. injection of the GUCY2C-specific ligand heat-stable enterotoxin (ST) induced phosphorylation of VASP in the hypothalamus at serine 239, which is cGMP dependent, but not at serine 157, which is cAMP dependent (*P<*0.001; Figure 4c). Furthermore, i.v. ST increased hypothalamic expression of c-Fos (*P<*0.01) and POMC (*P<*0.05) in *Gucy2c*^*+/+*^, but not in *Gucy2c*^*-/-*^, mice (Figures 4d and e). The transcription factor c-Fos promotes POMC gene transcription,^24, 25^ and POMC is a key anorexigenic neuropeptide precursor in ARC signaling (Supplementary Figure S2).^26, 27^ These signaling events in the hypothalamus complement the anorexigenic effects produced by i.v. ST (*P<*0.01; Figure 4f). Importantly, unlike its effects on intestinal uroguanylin, DIO did not reduce the expression of hypothalamic GUCY2C (Figures 5a and b). Rather, hypothalamic GUCY2C mRNA (*P<*0.05) and protein (*P<*0.001) levels were higher in DIO mice compared with lean controls. Similar to uroguanylin expression in intestine, this effect of DIO on GUCY2C is reversible, and hypothalamic receptor levels were restored to normal levels in obese mice switched to a low-calorie diet (GUCY2C protein, *P<*0.01; Figures 5c and d). In DIO mice, i.v. ST increased hypothalamic VASP phosphorylation that occurred earlier and was greater as compared with lean mice (*P<*0.001; Figure 5e). This increase in a reporter of GUCY2C stimulation mirrored the increase in expression of GUCY2C itself (Figure 5f). Moreover, anorexigenic signaling by hypothalamic GUCY2C was preserved in DIO and i.v. ST reduced food intake in obese mice (*P<*0.01; Figure 5g).

### Transgenic uroguanylin reduces food intake, weight gain and comorbidities in DIO

**Figure 6:**
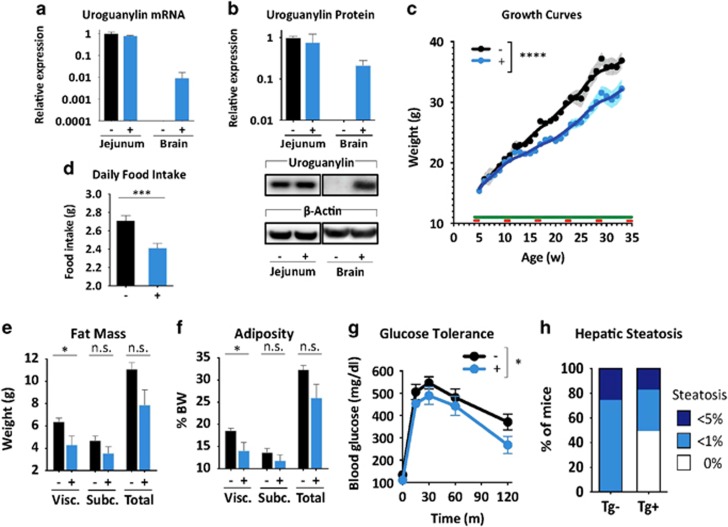
Transgenic uroguanylin opposes DIO-induced food intake, weight gain and metabolic comorbidities. (**a**, **b**) Uroguanylin mRNA and protein quantified by quantitative real-time PCR (qRT-PCR) and immunoblot and normalized to *Actb* or β-actin in transgene-control (*Camk2aCre-ER*^*T2*^; (−)) and transgene-positive (*Camk2aCre-ER*^*T2*^-*Rosa-STOP*^*loxP/loxP*^*-Guca2b*); (+)) mice (*n*=4 per group) following 5 days of i.p. tamoxifen (100 mg kg^−1^ per day). (**c**) Growth curves of ((−), *n*=5) and ((+), *n*=11) mice assembled from weekly weight measurements. From ~4 weeks of age, mice were maintained on the high-fat diet (green line) and injected with tamoxifen (100 mg kg^−1^ per day) for 5 days every 6 weeks (red dashes). Data were used to fit LOWESS (LOcally WEighted Sacatterplot Smoothing) curves, with s.e.m. as error envelopes. After 30 weeks, mice (*n*=4 (−), *n*=6 (+) mice) were assessed for (**d**) daily food intake; (**e**, **f**) visceral, subcutaneous and total adiposity, in mass (g), and in proportion (%) of total body weight; (**g**) glycemic control, determined by i.p. glucose tolerance tests and quantification of glucose area under the curve (AUC); and (**h**) hepatic steatosis. Severity of steatosis is related to the proportion (%) of hepatocytes with macrovesicular lipidosis. *P<0.05, ***P<0.001, ****P<0.0001, NS, not significant.

Uroguanylin loss with preservation of receptor sensitivity in DIO suggests that hormone replacement could reconstitute GUCY2C-mediated satiety. Local i.c.v. delivery of uroguanylin to the brain activated hypothalamic GUCY2C, recapitulating satiety responses produced by i.v. ligands^7, 8^ (*P<*0.0001; Supplementary Figure S3). Thus, mice were generated with a uroguanylin transgene under the control of a brain-specific tamoxifen-inducible promoter (Figures 6a and b) in order to target hypothalamic, but not intestinal, GUCY2C without the necessity of peptide delivery. Cohorts of mice with (+) and without (−) the transgene received the high-fat diet starting at 4 weeks of age, and body weights followed for ~30 weeks (Figure 6c). By week 12, the difference in cumulative weight gain of (−) and (+) mice was 3.76 g (95% confidence interval (CI) 2.34–5.17 g, *P*<0.001) or 24.43% (95% CI 15.24–33.61%, *P*<0.001) of their baseline weight (Supplementary Table S3). By week 24, the difference in cumulative weight gain was 5.63 g (95% CI 2.09–9.17 g, *P*<0.01) or 36.61% (95% CI 13.59–59.63%, *P*<0.01; Supplementary Table S3). Reduced weight gain was associated with a difference in daily food consumption between (−) and (+) mice of 0.30 g (95% CI 0.14–0.45 g, *P*<0.001) or 10.97% (95% CI 5.26–16.68%, *P*<0.001) after 30 weeks on a high-fat diet (Figure 6d). Stool water content was identical in (−) and (+) mice, confirming that central expression of transgenic uroguanylin did not induce intestinal GUCY2C fluid secretion (Supplementary Figure S4). However, comorbidities of obesity were lower in (+) compared with (−) mice including visceral adiposity (*P*<0.05; Figures 6e and f), glucose intolerance (*P<*0.05; Figure 6g) and hepatic steatosis (Figure 6h and Supplementary Figure S5).

## Discussion

The obesity pandemic continues to grow unabated and will soon be the leading cause of morbidity and mortality worldwide, highlighting the unmet medical need for new disease management paradigms.^1, 2, 3, 4, 5^ In the context of this burgeoning clinical need, there is a paradoxical gap in understanding molecular mechanisms contributing to the pathophysiology of obesity that can be targeted for therapy. The uroguanylin-GUCY2C gut–brain axis has emerged as a regulator of feeding, energy homeostasis, body mass and metabolism in normal physiology in rodents.^6, 7, 8^ Moreover, like mice, the consumption of nutrients induces endocrine secretion of uroguanylin into the circulation as the afferent limb of a gut–brain axis in humans as well.^8^ The present studies reveal that DIO reduced intestinal uroguanylin expression and eliminated its postprandial secretion into the circulation. In contrast, hypothalamic GUCY2C expression and sensitivity was increased in DIO, with preserved satiety responses induced by cognate ligands. Indeed, hormone replacement with transgenic uroguanylin expressed in brain restored anorexigenic signaling, opposing the development of obesity and its comorbidities. These observations suggest a previously unanticipated mechanism contributing to the pathophysiology of obesity in which DIO disrupts the uroguanylin gut–brain endocrine axis regulating feeding, energy homeostasis and metabolism. However, preservation of hypothalamic GUCY2C in DIO suggests the feasibility of a therapeutic paradigm involving cognate ligand replacement to manage obesity and its comorbidities.

DIO reduced uroguanylin mRNA and protein in intestine, associated with a commensurate reduction in VASP phosphorylation, a reporter of GUCY2C-cGMP signaling.^15^ Similarly, DIO eliminated postprandial increases in serum uroguanylin, reducing endocrine satiety signals to the hypothalamus.^8^ DIO-induced uroguanylin loss was mediated by ER stress, reflected by increases in the canonical marker BiP.^21^ Furthermore, tunicamycin, which induces ER stress, mimicked DIO and reduced uroguanylin, whereas TUDCA, a chemical chaperone that relieves ER stress, restored uroguanylin in DIO.^22^ In that context, metabolic disruption associated with glucolipotoxicity induces ER stress in pancreatic β-cells, leading to degradation of insulin mRNA and inhibition of insulin biosynthesis.^32, 33^ In fact, insulin and uroguanylin share characteristics making them particularly susceptible to ER dysfunction: both are synthesized as prepropeptides, dependent on proper folding for function and secreted as endocrine hormones.^34, 35^ Importantly, the effects of DIO on hormone expression were reversible, and returning mice on a high- to a low-fat diet restored intestinal uroguanylin expression.

Suppression of uroguanylin expression appears to be mediated by ingested calories, rather than the pathophysiological milieu of obesity. Although chronic overnutrition with high-fat or high-carbohydrate diets suppressed uroguanylin expression, cohorts maintained on the high-fat diet developed obesity whereas those on the high-carbohydrate diet maintained lean body weights. Furthermore, reversing chronic overnutrition from a high-fat diet by restricting calories restored uroguanylin levels in wild-type and *ob/ob* mice, even though they remained obese. These observations reveal uroguanylin loss in the absence of obesity, and hormone recovery despite the persistence of obesity, respectively. They suggest that similar to pancreatic β-cells and insulin,^32, 33^ overnutrition itself induces ER stress resulting in uroguanylin loss. From these observations, it is tempting to suggest a management strategy in obese patients in which calorie restriction restores intestinal uroguanylin expression to reconstitute the GUCY2C gut–brain axis that, in turn, creates a positive feedback loop improving satiety, body weight and metabolism.

GUCY2C is expressed in hypothalamus^6, 8^ and i.v. and i.c.v. GUCY2C ligands mediate central satiety responses regulating feeding in *Gucy2c*^*+/+*^ mice^7, 8^ but not *Gucy2c*^*-/-*^ mice.^8^ The hypothalamus, which regulates homeostatic processes including energy balance, encompasses nuclei with discrete functions.^36^ Among nuclei that regulate energy homeostasis, the ARC is a primary integrator of neural and peripheral inputs, including energy signals delivered by the circulation.^37, 38^ In turn, the output of the ARC includes orexigenic and anorexigenic neuropeptides, including POMC.^39, 40^ Here, we reveal that GUCY2C is enriched in the ARC. Furthermore, i.v. uroguanylin induces GUCY2C-specific expression of POMC in the hypothalamus, a response primarily restricted to the ARC (see Supplementary Figure S2).^39, 40^ Moreover, these molecular responses are associated with the induction of satiety by i.v. and i.c.v. uroguanylin.^7, 8^ These observations for the first time provide a topographic and mechanistic basis for the regulation of feeding by hypothalamic GUCY2C.

In contrast to uroguanylin, expression of hypothalamic GUCY2C is preserved in DIO mice. Indeed, i.v. ST increased hypothalamic VASP phosphorylation in DIO mice, revealing the persistent responsiveness of hypothalamic GUCY2C to endocrine stimulation in chronic overnutrition. Moreover, i.v. ST induced satiety in DIO mice, demonstrating that endocrine sensitivity in the hypothalamus is preserved in the context of a chronic high-fat diet. Given the role of uroguanylin in opposing DIO,^6, 7, 8^ and the potential role of hypothalamic GUCY2C in mediating these responses,^8^ the present study suggests a novel pathophysiological paradigm contributing to calorie-induced obesity and its comorbidities. Thus, although chronic overnutrition resulting in DIO produces hormone insufficiency in the endocrine ‘gland', reducing intestinal uroguanylin expression, the function of the receptor GUCY2C at the ‘end-organ', the hypothalamus, is amplified. In turn, this model of classical endocrine insufficiency should be amenable to reconstitution by hormone replacement. Indeed, transgenic uroguanylin expressed in the brain reduced food consumption and opposed obesity and its comorbidities in mice on a chronic high-fat diet. Importantly, these effects were enduring, persisting undiminished for up to 24 weeks, suggesting an absence of desensitization of GUCY2C or its downstream signaling events by transgenic hormone replacement.^41^ These observations highlight the potential for hormone replacement with GUCY2C ligands for durable management of DIO and its comorbidities.^6, 7, 8^

Targeting GUCY2C for antiobesity therapy is particularly appealing because it leverages a novel endogenous endocrine axis. Although the endogenous circuit is damaged, the impairment is a hormone deficiency with preservation of receptor function, and therapeutic strategies for hormone replacement are conceptually straightforward. This is in sharp contrast to the anorexigenic endocrine hormones leptin and insulin, whose serum concentrations rise in DIO, leading to chronic overstimulation and cognate receptor desensitization, resulting in loss of efficacy of these hormone receptor axes.^42, 43^ Indeed, obesity is pathognomonically associated with chronic hyperleptinemia and hyperinsulinemia and hormone overexpression with receptor desensitization has challenged efforts to employ these impaired satiety circuits as therapeutic targets. These examples underscore the importance of ensuring the integrity of the signaling system in therapeutic strategies targeting obesity. Here, preserved GUCY2C satiety signaling defines a system compatible with a strategy of therapeutic hormone replacement.

Although uroguanylin has emerged as the key afferent limb of a novel gut–brain axis regulating body mass, the precise central mechanisms and efferent pathways mediating these effects continue to be defined. In one study, i.c.v. uroguanylin failed to regulate appetite.^6^ However, these studies used rats, a model for which there is no experience in pharmacokinetic or pharmacodynamic parameters of uroguanylin with respect to feeding, energy homeostasis or body mass. More recently, uroguanylin delivered by constant i.c.v. infusion for 7 days produced weight loss in DIO mice that was independent of feeding but rather reflected increases in vagus nerve-dependent fecal output and brown adipocyte metabolic rate.^7^ Continuous supraphysiological concentrations of uroguanylin (~10^−4^–10^−5^ M),^44^ in vast excess of the Kd of hormone-receptor binding (~10^−9^ M), likely induced long-term desensitization of GUCY2C and its efferent regulation of feeding.^41^ Supraphysiological concentrations of uroguanylin could induce novel GUCY2C-independent effects, either through additional guanylyl cyclases expressed in the brain^45^ or through receptors other than guanylyl cyclases.^6, 46^ These observations stand in contrast to transgenic uroguanylin replacement here that produced hormone levels that induced durable anorexigenic responses opposing weight gain for at least 24 weeks without desensitization or alterations in the metabolic rate.^8^ These considerations highlight the importance of defining efferent mechanisms and central receptors that mediate them to maximize therapeutic opportunities for obesity and its comorbidities represented by the uroguanylin gut–brain axis.

Loss of intestinal uroguanylin expression with chronic overnutrition mirrors loss of intestinal GUCY2C hormones in colorectal tumorigenesis.^47^ It is tempting to speculate that this represents one molecular basis for the established association between obesity and colon cancer,^48, 49^ and highlights the role of GUCY2C agonism underlying homeostatic physiology. Thus, reconstitution of GUCY2C signaling by hormone replacement could restore anorexigenic responses corrupted by DIO, reducing obesity and its comorbidities. In turn, reduction of caloric intake could allow recovery of endogenous GUCY2C ligand expression, promoting GUCY2C agonism opposing both DIO and intestinal tumorigenesis. The implications for translation of these observations into improvements in patient care underscore the importance of future studies to address this compelling hypothesis.
