# Supplementary material for: Calorie-induced ER stress suppresses uroguanylin satiety signaling in diet-induced obesity
Source: Nutr Diabetes. 2016 May 23;6(5):e211–. doi: 10.1038/nutd.2016.18 (PMC4895379; doi:10.1038/nutd.2016.18)

Supplementary Figure Legends

Supplementary Figure S1. Negative controls for uroguanylin immunofluorescence staining. Jejunum from mice maintained on low- or high-calorie diets were stained as described in Figure 1C, except primary anti-uroguanylin antibody was omitted. Sections were counterstained for β-catenin (red) and nuclei (blue).

Supplementary Figure S2. Expression of NPY and POMC mRNA quantified by RT-PCR, in nuclei microdissected from the hypothalamus (Hypothal) of wild type mice. ARC: arcuate nucleus; LPN: lateral preoptic nucleus; MPN: medial preoptic nucleus; SCN: suprachiasmatic nucleus; MN: mammillary nucleus; PMN: premammillary nucleus.

Supplementary Figure S3. ICV administration of uroguanylin induces satiety. Food intake following ICV injection with 10 μg of TJU or prouroguanylin (GUCA2B), $n = 4$ per group. **** $P < 0.0001$.

Supplementary Figure S4. Transgenic uroguanylin expressed in brain does not affect stool water content. Stool collected from both groups of mice in Figure 6C (4-6 mice per group) at 30 wk was dried and weighed to calculate % water content. n.s., not significant.

Supplementary Figure S5. Representative hepatic steatosis in mice after 30 weeks on a high-fat diet with (+) and without (-) transgenic uroguanylin (Tg).

SUPPLEMENTARY TABLES

Supplementary Table S1. Mouse diet calorie and nutrient content.

**Calorie Content**	**Lean** (Diet 5010)	**High Carbohydrate** (Diet 58Y1)	**High Fat** (Diet 58Y2)
**Protein (% energy)**	28.8	18.0	18.1
**Fat (% energy)**	12.7	10.2	61.6
**Carbohydrate (% energy)**	58.5	71.8	20.3
**Energy (kcal/g)**	3.08	3.80	5.10

Supplementary Table S2. Changes in body weights with different diets.

Cohort	Week	Mean Body Weight (g)	SEM	*n*
Low	14	29.79	0.9603	10
High		48.70	0.5481	10
Mod		31.30	1.3030	10
Low	14	32.07	0.7178	10
High		52.28	0.8315	10
Rev		52.14	0.9697	16
Low	18	31.50	0.8434	10
High		51.96	0.6786	10
Rev		40.80	1.2090	16
WT	0	25.68	0.5850	6
Ob-AL		48.80	0.9524	5
Ob-R		47.20	1.1501	6
WT	6	28.63	0.5457	6
Ob-AL		59.24	3.8305	5
Ob-R		41.97	0.6469	6

Supplementary Table S3. Changes in body weights with transgenic uroguanylin expression.

**Changes in Weight at Week 12 and Week 24**		
	**Tg-**	**Tg+**
**Weight**		
n	4	11
Baseline mean (SEM) (g)	15.38 (0.789)	15.37 (0.331)
	**Week 12**	
n	4	11
Mean (SEM) (g)	26.85 (0.466)	23.09 (0.384)
Change from baseline (SEM) (g)	11.48 (0.466)	7.72 (0.384)
Difference from Tg- (95% CI) (g)		3.76*** (2.34-5.17)
Change from baseline (SEM) (%)	74.63 (3.033)	50.21 (2.500)
Difference from Tg- (95% CI) (%)		24.43*** (15.24-33.61)
	**Week 24**	
n	4	6
Mean (SEM) (g)	37.20 (1.023)	31.57 (1.136)
Change from baseline (SEM) (g)	21.83 (1.023)	16.19 (1.136)
Difference from Tg- (95% CI) (g)		5.63** (2.09-9.17)
Change from baseline (SEM) (%)	141.95 (6.654)	105.34 (7.388)
Difference from Tg- (95% CI) (%)		36.61** (13.59-59.63)

SUPPLEMENTARY EXPERIMENTAL PROCEDURES

ICV uroguanylin-induced satiation

C57BL/6J mice with a cannula surgically implanted into the third ventricle were purchased (The Jackson Laboratory), acclimated to individual cages with wire-mesh floors and Diet 58Y1 for 1 week, fasted for 16 h overnight, and lightly anesthetized with isoflurane for insertion of injector cannulae into guide cannulae. Five µl (10 µg) of TJU or prouroguanylin were injected at 1 µl/min for 5 min. Mice were returned to their individual cages and provided Diet 58Y1. Consumption was measured 1, 2, and 4 h following peptide injection and re-feeding.

Supplementary Figure S1

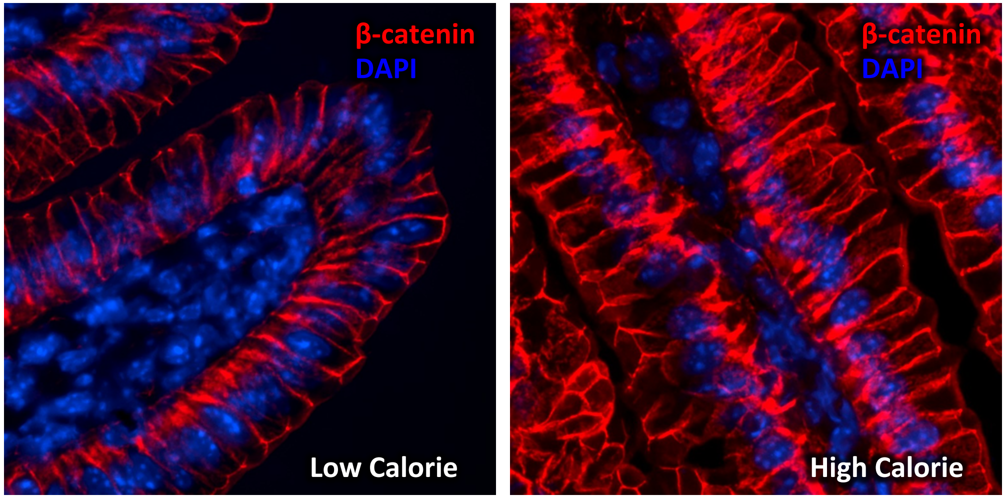

Supplementary Figure S2

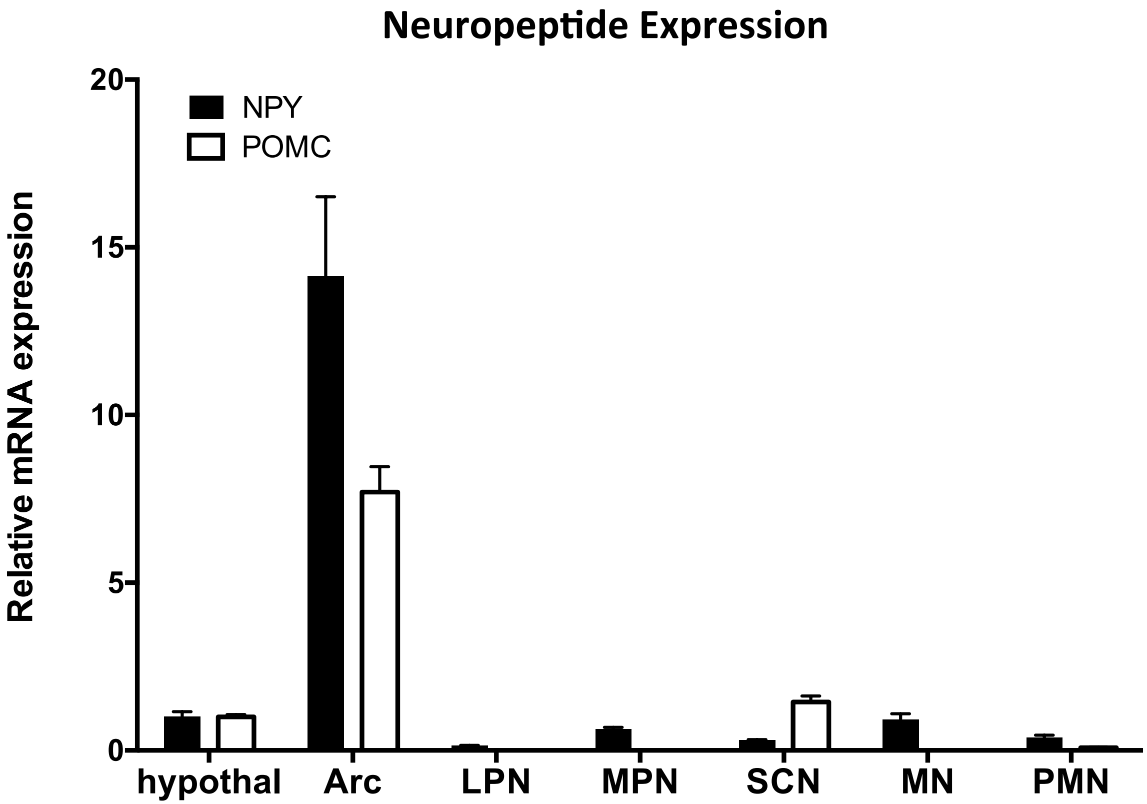

Supplementary Figure S3

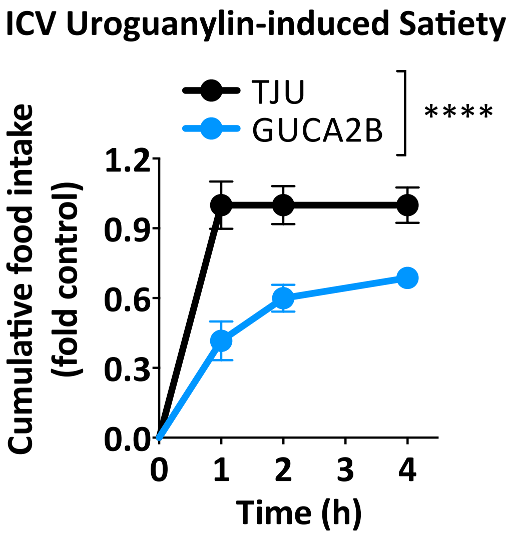

Supplementary Figure S4

A **Stool Water Content**

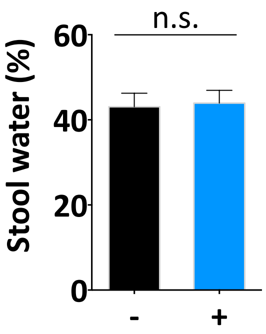

Supplementary Figure S5

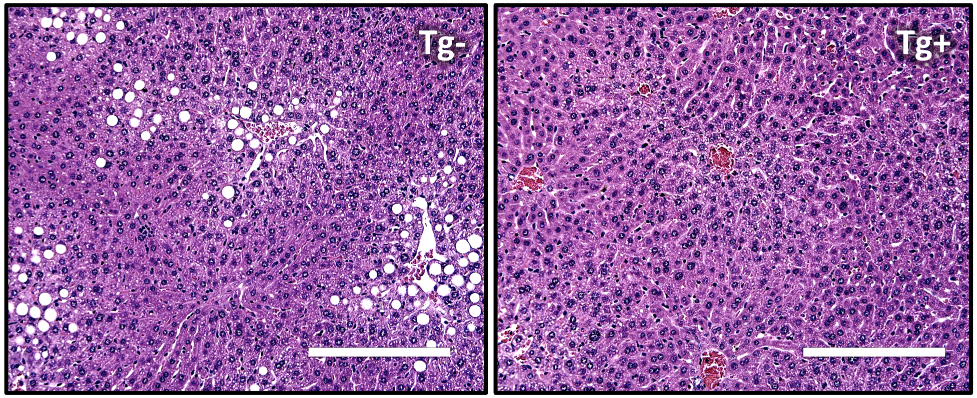

Supplement: Supplementary Information [file nutd201618x1.pdf]
